# A simple score derived from bone marrow immunophenotyping is important for prognostic evaluation in myelodysplastic syndromes

**DOI:** 10.1038/s41598-020-77158-z

**Published:** 2020-11-20

**Authors:** J. R. Vido-Marques, S. C. Reis-Alves, S. T. O. Saad, K. Metze, I. Lorand-Metze

**Affiliations:** 1 Department of Internal Medicine, Faculty of Medical Sciences, University of Campinas, Campinas, Brazil; 2 Hematology and Hemotherapy Center, University of Campinas, Carlos Chagas Street, 480, Campinas, São Paulo, 13083-878 Brazil; 3 Department of Pathology, Faculty of Medical Sciences, University of Campinas, Campinas, Brazil

**Keywords:** Biological techniques, Cancer, Medical research

## Abstract

Immunophenotyping of bone marrow (BM) precursors has been used as an ancillary diagnostic tool in myelodysplastic syndromes (MDS), but there is no general agreement about which variables are the most relevant for prognosis. We developed a parsimonious prognostic model based on BM cell populations well-defined by phenotype. We analyzed 95 consecutive patients with primary MDS diagnosed at our Institution between 2005 and 2012 where BM immunophenotyping had been performed at diagnosis. Median follow-up: 42 months (4–199). Median age: 67 years (33–79). According to IPSS-R, 71 cases were low or intermediate risk. Flow variables significant in the univariate Cox analysis: “%monocytes/TNCs”, “% CD16^+^ monocytes/TNCs”, “total alterations in monocytes”, “% myeloid CD34^+^ cells”, “number of abnormal expressions in myeloblasts” and “% of B-cell progenitors”. In the multivariate model remained independent: “% myeloid CD34^+^ cells”, B-cell progenitors” and “% CD16^+^ monocytes/TNCs”. These variables were categorized by the extreme quartile risk ratio strategy in order to build the score: % myeloid CD34^+^ cells” (≥ 2.0% = 1 point), B-cell progenitors” (< 0.05% 1 point) and “CD16^+^ monocytes/TNCs” (≥ 1.0% 1 point). This score could separate patients with a different survival. There was a weak correlation between the score and IPSS-R. Both had independent prognostic values and so, the flow score adds value for the prognostic evaluation in MDS.

## Introduction

Immunophenotyping of bone marrow (BM) precursors by multiparametric flow cytometry (FCM) has proven to be a useful ancillary tool for the differential diagnosis of myelodysplastic syndromes (MDS) and unexplained non-clonal cytopenias^1–10^. Besides, several studies aimed to investigate the prognostic value of flow cytometric (FCM) features, especially those which could add independent information to the established clinical scores, such as IPSS-R, but at the moment there is no general agreement about which are the most relevant prognostic flow variables^8,11–15^. This is mainly due to the large variety of FCM features examined among different studies^4,7–14^, some of them examining small patient cohorts or short follow-up times^4,8,9,15^. Furthermore, some quantitative FCM features, such as the mean fluorescence intensity of antigen expressions are difficult to interpret, since they depend on comparisons with normal values, which must be individually standardized for each laboratory.

Some prognostic scores based on FCM parameters have already been proposed, such as the Ogata score^5,6^, the Wells’ FCSS^8,9^ and the Red Score^13^ but they are not widely used in daily practice, in part because they are laborious and based on a large number of markers, which are difficult to standardize and depend on highly trained operators with good expertise^9,10,15^. The most used is the Ogata score^6^, that carries two parameters with a known prognostic significance, namely the “myeloblast-related cluster” and the “B-cell progenitor-related cluster”, with a known relation to patients’ prognosis, together with “Lympho/Gran CD45 ratio”, “Gran/Lympho SSC ratio” that have not shown a relevant meaning for prognosis. Therefore, this score is used predominantly for differential diagnosis between MDS with a normal karyotype and non-clonal peripheral cytopenias.

In this context we tried to create a prognostic model based on flow cytometric parameters which would be parsimonious, based only on few easily reproducible variables and which would add significantly new information to the already established IPSS-R score. The design was an uni-Institutional prospective patient cohort study with a long follow-up. Especially, we have addressed the distribution of the subsets of monocytic precursors in BM based on their expression of CD14 and CD16. The importance of these subsets in peripheral blood has been recently shown for the diagnosis of chronic myelomonocytic leukemia (CMML)^16,17^. However, their distribution in BM has not been studied in detail.

## Methods and development of the score

### Patients

The present study includes consecutive patients with primary MDS diagnosed at our Institution between 2005 and 2012. Diagnosis was made by WHO 2008 criteria based on clinical data, PB counts, BM cytology and histology as well as cytogenetics^18^. BM immunophenotyping was performed during the diagnostic work-up. Deficiency anemias, viral infections and autoimmune disorders had been excluded. For all cases IPSS-R was assessed^19^. Overall survival of the patients was calculated from diagnosis until death or last follow-up for patients receiving only supportive care. Patients eligible for cytotoxic therapy or bone marrow transplantation were censored at the time of the start of therapy.

### Flow cytometric analysis

Flow cytometric analysis was performed in BM collected in EDTA and diluted to a concentration of 5–7 × 10^6^ cells in 100 µl. Samples were processed within 24 h after sample collection. A standardized stain/lyse/wash protocol was used to study antigenic expression of the myelomonocytic series and CD34^+^ cell subsets. Details had been previously described^11^. Immediately after staining, samples were acquired in a FACSCalibur flow cytometer (Becton Dickinson—BD Biosciences, San José, CA, USA) using the CellQuest software (BD Biosciences). Information of least 100,000 events was acquired. The following antibody combinations were used: CD64/CD14/CD45/HLA-DR; CD16/CD11b/CD45/CD13; CD13/CD117/CD45/CD34; CD19/CD10/CD45/CD34 and CD7/CD56/CD45/CD34.

Diagnostic flow files were reanalyzed in the Infinicyt 1.7 version (Cytognos SL, Salamanca, Spain). In granulocytic precursors we assessed hypogranularity (SSC of granulocytes/SSC lymphocytes ratio, according to Ogata^6^) and decrease or increase (1 standard deviation from normal) in expression of CD11b, CD13 and CD16. Besides, the percentages of all monocytes, those of classical (CD14^+^/CD16^−^) and of CD16^+^ monocytes among total nucleated cells and their proportion among all monocytes^17^, as well as decrease in expression of HLA-DR, CD11b, CD14 and CD64 were examined. Cross-lineage expressions of CD56 and CD7 in granulocytes and monocytes (20% of the cells) were also assessed^2,10,11^. Antigen expression was compared to those of 15 controls obtained from BM aspirated from donors for bone marrow transplantation and analyzed with the same flow protocol as the patients.

Concerning CD34^+^ cells, we quantified the myeloid progenitors (CD13^+^ and/or CD117^+^), and decrease in expression (1 standard deviation of normal MFI) of CD13 or CD117 as well as aberrant expression of CD56 or CD7 in > 20% of the cells (until 4 alterations) and the percentage of B-lymphoid progenitors (CD34^+^/CD19^+^/CD10^+^). All percentages of positive cells were computed among total nucleated cells (TNCs). Values for myeloid CD34^+^ cells were considered normal when below 2%^6^. For hematogones (H1), the normal Brazilian reference values for age were used^20^.

### Statistical analysis

First, descriptive statistics was performed. Differences among groups and relations between phenotypic features and other risk factors were analyzed by non-parametric tests (Mann–Whitney and Kruskall–Wallis test, Spearman’s and Kendall´s rank order correlations).

Survival analyses were made using the Kaplan–Meier-diagram followed by the log-rank test and uni- and multivariate Cox regressions with the backward conditional strategy for variable selection, considering *p* = 0.05 for input and *p* = 0.1 for output, including all variables with *p* < 0.1 in the univariate models.

The internal stability of the models was tested by bootstrap resampling^21–23^. In brief, 100 new data sets with the same size of the original one, were created by random sampling with replacement. Cox regressions with the same conditions as in the original data set were performed for each of these new data sets.

**Figure 1:**
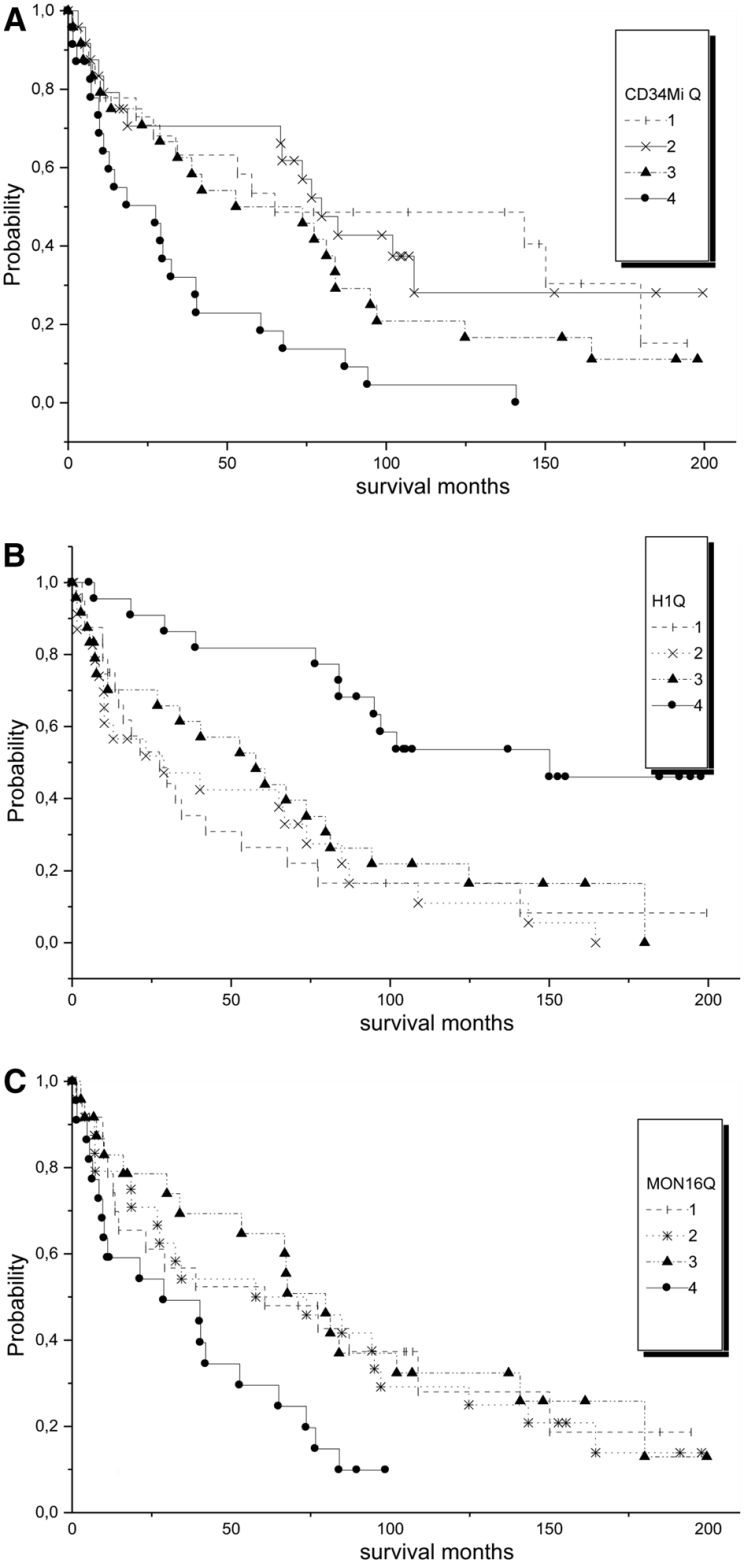
Kaplan–Meier survival analysis of the three variables used to construct the flow score, separating patients by quartiles. (**A**) myeloid progenitors. Values for quartile 1: median and range: 0.1(0.02–0.2); for quartile 2: 0.35 (0.24–0.64); for quartile 3: 1.05 (0.68–2.01) and for quartile 4: 5.07 (2.22–10.48). The patients presenting values in the upper (4) quartile (> 2%) had a significant worse survival. *p* = 0.001; (**B**) B-cell progenitors. Values for quartile 1: median and range: 0.0 (0.0–0.0); for quartile 2: 0.0 (0.0–0.0); for quartile 3: 0.01 (0.0–0.04) and for quartile 4: 0.19 (0.05–0.72). Patients presenting values > 0.05% (upper quartile) had a better survival. *p* < 0.0001. (**C**) CD16^+^ monocytes/TNCs. Values for quartile 1: median and range: 0.09 (0.0–0.15); for quartile 2: 0.19 (0.16–0.35); for quartile 3: 0.63 (0.35–0.96) and for quartile 4: 1.44 (0.99–10.5). Patients with values > 1.0% had a worse survival. *p* = 0.05.

For the prognostic flow cytometric variables which remained as independent prognostic factors in the final multivariate model (with *p* < 0.0001) (“% CD16^+^ monocytes”, “myeloid CD34^+^ cells” and “H1”), we defined cut-points by using the criteria of the extreme quartile risk ratio^24,25^ in order to build a flow score. For that purpose, the values for the quartiles of each variable were assessed, and examined by the Kaplan–Meier method followed by the log-rank test, to see which cut-point could separate best patients with a different survival (Fig. 1). Then, one point was given for each variable in the range of a worse survival. Finally, the score was tested by the Kaplan–Meier method to see if it was able to separate patients with a different survival.

In order to compare IPSS-R and our flow score, we used the AKAIKE information criteria (AIC), which are based on information theory^26^. When a mathematical (idealized) model represents a set of true data from real life, this representation will never be exact. Therefore, some information will be lost by using the calculated model. The Akaike information theory estimates this relative information loss. Good models are characterized by minimal information loss. To apply AIC in practice, we start with a set of candidate models, and then find the models' corresponding AIC values. Most of the times there will be loss of information as the candidate models represent the "true model," i.e. the process that generated the data. The Akaike information criterion takes into consideration both the simplicity and goodness of fit of the model.

Our aim was to select the model that minimizes the information loss among the candidate models. We could not choose with certainty, but we could minimize the estimated information loss. We calculated the so-called Akaike weights which permit the simultaneous comparison of various candidate models.

SPSS 15.0 and Winstat softwares were used for calculations.

### Ethical approval

All methods were performed according to the regulations of the Brazilian Commission for Ethics in Research (CONEP) and the Helsinki Declaration. Informed consent was obtained from all the participating patients. The project had been approved by the Ethics Committee (ERC) of the University of Campinas (Proc 0652.0.146.000-08).

## Results

A total of 101 patients with newly diagnosed MDS entered the study. Among them, 6 had a follow-up less than one month and were excluded. So, the study was based on 95 patients. Their flow data were compared with those of 13 BM donors for transplantation.

The median time of observation was 42 months (4–199 months). At the end of the observation, only 23 patients remained alive. The characteristics of the patients are shown on Table 1. According to the WHO classification, the majority of the patients had refractory cytopenia with multilineage dysplasia. According to IPSS-R only 24 cases were high or very high risk.

**Table 1:** Demographic features of the patients.

	Controls n = 15	MDS n = 95
Age (years)	40 (31–52)	67 (33–79)
Male/female	9/6	62/33
IPSS-R		
Very low risk		13
Low risk		34
Intermediate		24
High risk		17
Very high risk		7
WHO types		
Refractory anemia		6
5q-syndrome		2
RCMD*		61
RAEB I**		12
RAEB II**		14

*Refractory cytopenia with multilineage dysplasia, **Refractory anemia with excess of blasts.

Regarding the flow cytometric variables (Table 2), in the granulocytic lineage, SSC was decreased compared to normal controls (< 6.9) in 46 cases. Besides, 4 cases had no phenotypic alterations, 32 had one, 28 had two, 27 had three and 4 cases had four alterations.

**Table 2:** Phenotypic features of the patients compared to the controls (median and 10–90% percentiles). MDS cases are divided between those with normal and increased BM monocytes.

	Controls N = 15	MDS with normal percentage of monocytes n = 69	MDS with increased monocytes n = 26	*p* values***
SSC Gran/lympho ratio	8.7 (6.9–9.5)	7.3 (4.7–9.6)	6.5 (4.6–9.2)	0.009
% total monocytes	3.7 (2.0–5.6)	1.9 (0.5–4.4)	9.1 (5.7–19.1)	< 0.0001
% classical monocytes*	3.46 (1.8–5.1)	1.46 (0.39–3.55)	6.7 (4.5–16.8)	< 0.0001
% classical monocytes**	92% (83–95)	85% (60–95)	84% (53–97)	0.52
% CD16^+^ monocytes*	0.29 (0.1–0.5)	0.19 (0.08–1.0)	1.1 (0.30–4.7)	< 0.0001
% CD16^+^ monocytes**	7.6 (4.9–16.8)	15% (5–40)	15% (3–45)	0.49
% B-cell progenitors*	0.18 (0.05–0.58)	0.0 (0.0–0.23)	0.0 (0.0–0.08)	< 0.0001
% CD34^+^ myeloblasts*	0.7 (0.34–1.0)	0.78 (0.15–6.8)	0.26 (0.08–4.5)	0.008

*Among total nucleated cells (TNCs), **among total monocytes, *** comparing all 3 groups.

In 26 MDS patients the proportion of monocytes among all nucleated cells was increased compared to our control group (5.6% among TNCs—Table 2). There were high correlations between “% monocytes/TNCs” and “classical monocytes/TNCs” and CD16^+^ monocytes/TNCs” (r = 0.96; *p* < 0.0001 and r = 0.72; *p *< 0.00001 for CD16^+^ ones respectively in the Spearman’s correlations), but this correlation turned non-significant when calculated between “% monocytes/TNCs” and the proportion of each subtype among total monocytes. So, the proportions between classical and CD16^+^ monocytes were maintained independently of the total monocyte count. Concerning alterations in antigen expressions, 12 cases had no alterations, 26 showed one, 24 two, 32 three and one case had four alterations.

In 25 patients the percentage of myeloid CD34^+^ cells, was above 2%. Abnormal co-expressions among these ells, was present in 43 patients: 14 with one, 13 with two and 16 three aberrant expressions. Hematogones type I were not detectable in 54 cases and in 18 additional patients they were less than 0.05% among total nucleated cells. Only in 23 cases they were in the normal range for age.

### Survival

Table 3 shows all the variables that were significant in the univariate Cox regression. Concerning the flow variables, a multivariate Cox regression was run with: “% monocytes/TNCs”, “% CD16^+^ monocytes/TNCs”, “total alterations in monocytes”, “% myeloid CD34^+^ cells”, “number of abnormal expressions in myeloblasts” and “% of B-cell progenitors”. The variables remaining as independent in the models were: “% myeloid CD34^+^ cells”: B = 0.156; HR 1.167 (1.096–1.243); *p* < 0.0001; “% CD16^+^ monocytes/TNCs” B = 0.348; HR 1.416 (1.170–1.714); *p* < 0.0001 and B-cell progenitors”: B = – 7.068; HR 0.003 (0.000–0.126) *p* < 0.0001. In the bootstrap stability test, they were present in 70%, 68% and 88% respectively of the new models, while “% monocytes” was present in 10%, “total alterations in monocytes” 38% and “number of abnormal expressions in myeloblasts” in 28% of the new data sets.

**Table 3:** Hazard ratio and 95% CI for age IPSS-R and the flow variables used in the models.

	B***	HR (95% CI)	*p*
Age	0.024	1.024 (1.009–1.039)	0.002
IPSS-R	0.687	1.988 (1.583–2.497)	< 0.0001
% total monocytes	0.039	1.040 (0.992–1.090)	0.106
% classical monocytes**	–1.940	0.144 (0.032–0.645)	0.011
% CD16^+^ monocytes*	0.345	1.425 (1.192–1.702)	< 0.0001
% CD16^+^ monocytes**	1.944	6.98 (1.558–31.343)	0.011
Number of abnormal antigen expressions in monocytes	0.359	1.432 (1.142–1.795)	0.002
% CD34 + myeloid progenitors	0.156	1.169 (1.100–1.241)	< 0.0001
Number of abnormal antigen expressions in myeloid progenitors	0.237	1.289 (1.075–1.546)	0.006
% H1	–7.068	0.001 (0.00–0.039)	< 0.0001

*Among all nucleated cells; **among all monocytes; ***B = cumulative regression coefficient.

### Development of the prognostic score

In order to construct a score for practical application we categorized the three continuous variables according to the principle of the extreme quartile risk ratio and got the following suggestion:

- % myeloid CD34^+^ cells”—one point for values ≥ 2.0% (Fig. 1A).
- % of B-cell progenitors”—one point for values < 0.05% (Fig. 1B).
- CD16^+^ monocytes/TNCs—one point for values ≥ 1.0% (Fig. 1C).

The score is the sum of the three points and ranges between 0 and 3.

**Figure 2:**
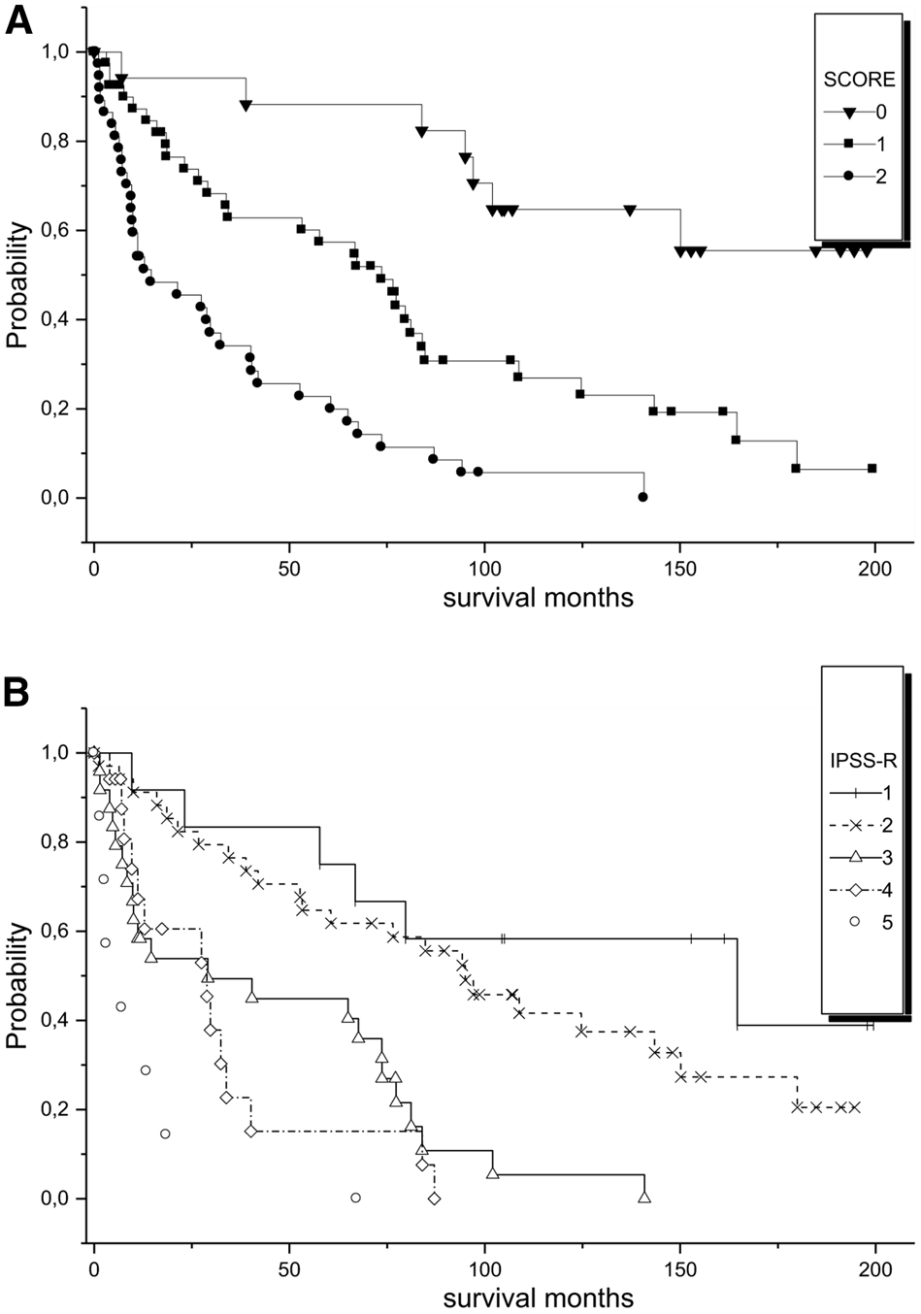
(**A**) survival analysis of the patients separated by the points of the score. The 2 patients with 3 points were added to the group with 2 points for the survival analysis in the Kaplan–Meier method. *p* < 0.0001. (**B**) the patients analyzed according to the IPSS-R categories: 1 = very low risk, 2 = low risk, 3 = intermediate risk, 4 = high risk and 5 very high risk. *p* < 0.0001.

Among all patients, 17 had 0 points, 40 had 1 point, 35 had 2 and 3 had 3 points. Our score separated well groups with different survival in the Kaplan–Meier method (Fig. 2A), as was also seen with IPSS-R (Fig. 2B).

There was a rather weak correlation between the flow score and IPSS-R: r = 0.305; p = 0.001 (Kendall´s tau correlation, significant at the 0.01 level, 2-tailed). Furthermore, in a multivariate Cox-model with IPSS-R and our flow score, both were independent variables for patients’ overall survival. In 100 bootstrap resampling sets of the original data, IPSS-R was present in 98% and the score in 100% of the models, thus showing the complementary nature of both scores, which is also demonstrated in Table 4. Comparing the three prognostic models in the original data and the resampling, the relative weights of the Akaike information criterion with the following data (mean, percentile 5 and 95): W IPSS-R = 0.0002 (0.000–0.0665), W flow score = 0.0008 (0.000–0.1837) and W IPSS-R + Flow Score = 0.9990 (0.7245–1.000). So, we can conclude that a model combining IPSS-R and our Flow Score describes much better overall survival of MDS patients than both scores separately. The flow score was able to add prognostic value to IPSS-R.

**Table 4:** Relation between the risk stratification of IPSS-R and the flow score.

IPSS-R	Score 0	Score 1	Score 2	Score 3
Very low risk	4	7	2	0
Low risk	10	16	8	0
Intermediate	2	6	15	1
High risk	1	7	8	1
Very high risk	0	4	2	1

## Discussion

In the present work we examined the prognostic relevance of several phenotypically well-defined hematopoietic precursor cell populations in a relatively large prospective uni-Institutional cohort of MDS patients with a long observation time, which permits a more reliable survival analysis. We also tried to construct a simple and reproducible score, which could be easily applied in daily practice. The ideal variables for this purpose should be easy to obtain and relatively independent of antibody combinations used and analytical conditions. These premises are fulfilled when quantifying well-defined cell subsets.

In our study, phenotypical alterations in granulocytic maturation had no influence on patients’ prognosis. Several of them had been included in other formerly described scores based on flow data^2,5,10^. These variables are difficult to reproduce, as they are based on the measure of mean fluorescence intensity of antigen expressions and therefore highly dependent on the type of equipment, the antibody fluorescence, and analysis software, so that comparisons with local control groups are necessary.

First, we confirmed the prognostic value of the increase in CD34^+^ myeloid progenitors and decrease of B-cell progenitors, which has already been described by our group^4,11,18^ as well as by others^1,4,6,7,10,11,14,16,18,27^. The number of phenotypic alterations in CD34^+^ myeloid progenitors were also associated with survival, but were less important as their total number in the multivariate Cox regression, and especially in the bootstrap stability test. A value > 2% for myeloid progenitors has been recognized as a good cut-point for malignancy, and included in the Ogata score^5,6^. It has also been claimed that this parameter is important in predicting progression to acute leukemia and overall survival of patients with MDS with IPSS-R intermediate risk^14^. In our study, we could confirm the importance of this variable on our patients’ overall survival. The cut-point for a worse survival proposed by the extreme quartile risk ratio (EQRR) was also “myeloid progenitors > 2%”.

Concerning B-cell progenitors (hematogones type I), using the same strategy we found that patients with hematogones within the upper quartile had a better survival. This value corresponded to the normal range for age in our population, as previously demonstrated in a Brazilian multicenter study^20^. Similar results had been described by other studies concerning MDS^28,29^ and ALL in remission after induction^30^. Although the decrease of hematogones is considered to be a diagnostic hallmark of MDS, a preserved number in low-risk cases is a sign of a better survival. This was found in 24% of our cases, and in 29% of the cases of low-risk MDS in the work of Chen et al^28^.

Furthermore, we demonstrated that the percentage of CD16^+^ monocytes among total nucleated cells, better than their percentage among all monocytes was associated with a worse survival. The proportion of classical monocytes varied proportionally to the total monocytes, and had only a weak influence on patients’ survival. The patients with increased CD16^+^ monocytes/TNCs, again in the highest quartile (> 1.0%) had a worse survival.

Recently, emphasis has been given to the distribution of monocyte subsets (classical, intermediate and non-classical) in peripheral blood based on their expression of CD14 and CD16^16,17^, for the diagnosis of chronic myelomonocytic leukemia (CMML) and its differential diagnosis with MDS presenting peripheral relative but not absolute monocytosis. Several studies have shown that in CMML, the proportion of classical (CD16^**-**^) monocytes are increased in CMML compared to cases of reactive monocytosis. In MDS, the values are very variable in peripheral blood^17^, but their distribution in BM has not been studied in detail. MDS cases with relative but not absolute peripheral monocytosis have been called oligomonocytic myelomonocytic leukemia^27,31^. Several of them progress to CMML or acute myeloid leukemia, so presenting a worse survival.

The association of some alterations in BM monocyte antigen expressions with the outcome of MDS patients have already been described in the first publications concerning BM immunophenotyping in MDS^9–12^, but only recently more attention has been drawn to number and type of antigen aberrancies. Recently, we have shown that the number of total BM monocytic precursors, as well as the increase in CD16^+^ ones (intermediate and non-classical) could be associated with a patients’ worse survival^11,12^. Therefore, we decided to study these parameters separately from antigenic aberrancies, and could confirm this finding.

So, we developed a “flow score” with the three variables “% myeloid CD34^+^ cells > 2%”, % of B-cell progenitors < 0.05%” and “CD16^+^ monocytes/TNCs > 1.0%” (one point for each)**.** The most frequent abnormality found was the decrease of B-cell progenitors. Increase in CD34^+^ myeloid progenitors and CD16^+^ monocytes were alterations found with a similar frequency. This score was able to separate groups of patients with a significantly different overall survival, independent and complementary to IPSS-R, and so, adding value to this clinical score. Stability tests were made for our cohort of patients, but the score should be validated in an independent cohort.

A wide variety of parameters generated by multiparametric flow cytometry of BM precursors in MDS have been examined for their diagnostic and prognostic importance^27^. Many works are retrospective studies and some have short observation times. Most of the flow abnormalities examined are based on “different from normal” variations in antigen expression, when compared to normal or reactive BM. All these aspects have hampered the standardization of the scores and the search for features that are able to add independent prognostic value to the clinical scores, especially to IPSS-R. So, we tried to construct a score based on the quantification of well-defined BM cell subsets, which is easier to standardize for clinical praxis. In our study, the flow variables related to quantification of specific cell subsets had a more robust prognostic significance than the variables related to antigen expressions. So, we used these variables to build the score.

The score developed in the present study was very robust to add additional prognostic information to IPSS-R. The Flow Score has several advantages: it is parsimonious, for it is based on only three cell types, well defined in several publications in the literature and is easily reproducible. Challenging the model by bootstrapping showed good intrinsic model stability. A test of external stability is however still missing and therefore reproducibility should be tested with a new cohort of patients, preferentially in another institution and population.
